# Nutritional Approaches to Managing Brain Fog: Insights Into Neuroinflammation, the Gut-brain Axis, and Sleep

**DOI:** 10.1007/s13668-026-00747-3

**Published:** 2026-04-10

**Authors:** Canan Altınsoy, Evrim Kahramanoğlu Aksoy, Seçkin Özgül, Derya Dikmen

**Affiliations:** 1 Department of Nutrition and Dietetics, Faculty of Health Sciences, Hacettepe University, Ankara, 06230 Türkiye; 2 Department of Nutrition and Dietetics, Faculty of Health Sciences, Recep Tayyip Erdogan University, Rize, 53020 Türkiye; 3 Department of Gastroenterology, Ankara Atatürk Sanatorium Training and Research Hospital, Ankara, 06230 Türkiye

**Keywords:** Brain fog, Neuroinflammation, Gut-brain axis, Gastrointestinal health, Sleep, Diet

## Abstract

**Purpose of Review:**

Brain fog is a common, poorly understood condition with symptoms like forgetfulness, mental slowness, difficulty concentrating, word-finding issues, and mental cloudiness. It disrupts daily life and reduces quality of life. The aim of this review was to examine the nutritional evidence that may influence the underlying pathophysiological mechanisms of brain fog; to assess both its potential benefits and current limitations in research, as well as to identify gaps in the literature to inform future studies.

**Recent Findings:**

Recent findings have increasingly highlighted the roles of neuroinflammation, dysregulation of the gut-brain axis, and poor sleep quality as key contributors to its pathophysiology. Diet and dietary components can influence brain fog by regulating inflammation, modulating gut microbiota, and affecting sleep quality. Anti-inflammatory diets contribute positively in this respect, whereas Western-style diets high in fat and sugar and rich in processed foods have a negative effect.

**Summary:**

The Mediterranean diet stands out in current research because its benefits are consistently documented across all three key domains, reducing neuroinflammation, supporting gut–brain communication, and improving sleep quality. By comparison, although ketogenic diets have plausible mechanistic support and encouraging in vitro and in vivo findings, the human evidence base remains limited, and outcomes are heterogeneous, precluding firm conclusions regarding efficacy across these domains. Referring individuals experiencing brain fog to a dietitian during this process is essential for providing individualised nutrition management. A nutritionally balanced diet that provides adequate energy and micronutrients and is rich in dietary fibre, antioxidants, and omega-3 fatty acids may support the management of brain fog. However, caution is warranted regarding probiotic supplementation; their use should be consistent with individual physiological needs and evidence-based practice, and the process should be managed by a qualified health professional.

## Introductin

The term ‘brain fog’ is increasingly used informally to describe difficulties in cognitive function. It refers to a broad but specific set of symptoms that affect cognition, particularly attention, memory, and language, as well as mood and fatigue [1, 2]. Patients have used this descriptive term to describe various cognitive complaints such as mental fuzziness, short-term memory loss, difficulty concentrating, and dizziness. Its roots trace back to the 19th century, Caraceni and Grassi (2011) [3, 4] suggest that the term probably derives from the German phrase “*Verdunkelung des Bewusstseins*”, meaning clouding or dimming of consciousness, first recorded in 1817. Over time, brain fog has been used synonymously with terms such as “clouding of consciousness” or “mental fog” [5]. In contemporary literature, however, it is understood as a type of temporary cognitive dysfunction characterised by a range of symptoms that impair mental functioning and often disrupt daily activities (Medical Genetics Identifier: MG:5131, Concept Unique Identifier: C0015676). Key symptoms include forgetfulness, mental slowness (or perceived slowing of processing speed), difficulty concentrating or focusing, trouble finding the right words, and a feeling that the mind is blank or “cloudy.” These symptoms most often manifest as problems with memory and attention, such as difficulties encoding and recalling information (e.g., words, names, stories, or numbers) and staying focused on a single thought. Individuals experiencing brain fog often describe their symptoms using various terms, reflecting a wide range of cognitive challenges [6]. Individuals experiencing brain fog may also report distractibility, forgetting intentions (e.g., the reason for entering a room), and struggling to switch between tasks [7].

Patients commonly describe “brain fog” as difficulty in maintaining attention and recalling information, often accompanied by fatigue and concerns about occupational performance. Reports also include, excessive daytime drowsiness and inattentive mistakes at work [8]. Additionally, many individuals describe a subjective sensation of feeling mentally slow, fuzzy, or spaced out, which significantly impairs their ability to think clearly or concentrate [9]. Brain fog has emerged as a prominent symptom of long-COVID syndrome [10]. Altough not a formally defined medical term [11, 12], the World Health Organization (WHO) refers to it as a colloquial expression for a common complaint of impaired cognitive function among patients experiencing post-acute COVID-19 [13]. Beyond COVID-19, brain fog has been been observed in many other medical conditions. It is documented in hypothyroid patients treated with levothyroxine (LT4) [14], in menopause [7], central disorders of hypersomnolence (such as narcolepsy and idiopathic hypersomnia), multiple sclerosis, lupus erythematosus [15], depression [16], and celiac disease [18]. Additionally, it is common in conditions like postural orthostatic tachycardia syndrome (POTS) [18], chronic fatigue syndrome, fibromyalgia [11], mastocytosis, autism spectrum disorders (ASD), and “mild cognitive impairment,” which is an early stage of Alzheimer’s disease [11]. Brain fog is also reported in patients undergoing or following chemotherapy, a phenomenon commonly referred to as “chemofog” or “chemobrain” [19, 20]. These findings demonstrate that brain fog is a widespread clinical symptom across a broad spectrum of medical conditions, from endocrine and autoimmune disorders to neurodegenerative and psychiatric diseases. This diversity underscores its clinical significance and highlights the complexity of the underlying mechanisms, which may involve multifactorial interactions such as inflammation, neuronal dysfunction, disruptions in the gut-brain axis, and metabolic disorders. Aside from the complex mechanisms contributing to brain fog and its links to various medical conditions, research suggests that certain risk factors may also influence the occurrence of this cognitive symptom cluster [21]. Female sex has been identified as a notable risk factor, with studies reporting a higher prevalence of brain fog among women [9, 21–23]. For instance, Jensen et al. (2022) highlighted that neurological and neuropsychiatric symptoms, including brain fog, are more commonly reported in women [24], while Asadi-Pooya et al. (2021) found that women are 1.4 times more likely to experience brain fog [9]. This finding is further supported by Gorenshtein et al. (2024), who conducted a systematic review and meta-analysis, concluding that women are at a higher risk for developing brain fog [22]. These studies collectively emphasise the role of female sex as a notable risk factor for brain fog in long COVID-19 and potentially other related conditions.

Currently, there is no specific treatment for brain fog. However, treatment approaches targeting cognitive function, mood disorders, sleep disturbances, and inflammation are suggested to potentially provide benefits [8, 23]. Clinical management typically adopts a multidisciplinary approach that incorporates cognitive rehabilitation, management of comorbidities, and anti-inflammatory interventions to address the underlying pathophysiology [8]. Within this framework, the present study critically examines the nutritional factors implicated in the pathogenesis of brain fog, with particular emphasis on inflammatory processes, gut-brain axis dysfunction, and sleep dysregulation. These elements will be assessed through a nutritional perspective, highlighting dietary interventions that may modulate their roles in cognitive impairment.

### Neuroinflammation and Diet

The exact aetiology of brain fog remains unclear; however, cellular mechanisms related to the central nervous system, particularly glial cell dysfunction associated with astrocytes and microglia [25, 26] are among the most commonly proposed underlying explanations [4, 27, 28]. Astrocytes, the predominant glial cells in the central nervous system (CNS), play a crucial role in regulating energy metabolism, supporting neuronal and synaptic activity, and maintaining the proper function and integrity of the blood-brain barrier (BBB) [29, 30]. Microglial cells, although not the primary component of the BBB, regulate its function and act as the principal defence in the central nervous system [31]. They play an important role in immune responses and maintenance of CNS homeostasis through close interaction with other glia cells, including neuronal cells, astrocytes and oligodendrocytes [32]. The first proposed hypothesis is that astrocyte infection leads to altered metabolic pathways, causing damage to neighbouring neurons that are supported by the infected astrocytes, ultimately resulting in astrocyte-associated glutamatergic dysfunction [33, 34]. Another theory suggests that activation of microglia leads to the release of pro-inflammatory molecules, an increase in oxidative stress, and mitochondrial dysfunction within microglia. When microglia and astrocytes are activated by stress, trauma, pathology, or infection, they secrete reactive oxygen species (ROS) and pro-inflammatory cytokines [35]. These neuroinflammatory responses and disrupted redox processes are believed to play significant roles in the progression of neurological symptoms, including brain fog [34]. An effective strategy for preventing brain fog is the regulation of peripheral and neuroinflammation by minimising risk factors and/or enhancing protective factors, such as maintaining a healthy diet throughout life [36]. One potential treatment approach involves reducing neuroinflammation through lifestyle modifications, starting with dietary changes, to address symptoms associated with brain fog. In their study, Krishnan et al. identified several potential causes of brain fog, including inadequately treated mood disorders, possible sleep disturbances, and changes in eating habits. They also emphasised the importance of referring patients experiencing brain fog to a dietitian for proper management [8]. Diet plays a significant role in inflammatory processes, which has been increasingly recognised in research. It is now well established that diet is linked to neuroinflammation, shedding new light on the importance of dietary choices in maintaining cognitive health [36, 37]. A poor-quality diet high in sugar, trans fats, and food additives but low in nutrient density has been linked to increased systemic inflammation and neuroinflammation [8]. Additionally, high-fat diets, Western-style eating patterns, macronutrient imbalances (whether due to deficiency or excess), improper carbohydrate and fat composition, inadequate micronutrient intake, and high salt consumption have all been associated with increased inflammation [38–40]. Considering the effect of diet on inflammatory processes, both food composition and the consumption of processed foods are essential factors. Pro-inflammatory diets are typically high in sodium because of excessive salt consumption and processed foods, while being low in potassium. This results in a reduced intake of potassium-rich foods like fruits and vegetables. Processed foods, which are often high in salt, added sugars, and saturated fats, further worsen inflammation. Therefore, it is recommended to reduce processed food intake and adopt healthier dietary patterns to mitigate these effects [38, 40]. Deficiency of certain vitamins, including B vitamins, vitamins C, D and E, can lead to neuroinflammation, which affects the normal functioning of neurons and homeostasis within the central nervous system [31, 41, 42]. Therefore, adopting an anti-inflammatory diet and addressing nutrient deficiencies are essential [41]. Such dietary approaches emphasise adequate and varied consumption of fruits (rich in polyphenols and antioxidants) and vegetables (dark leafy greens, colourful vegetables, etc.), while limiting processed food, refined carbohydrates and alcohol. They also recommend reducing the intake of red meat, high-fat dairy products, and saturated and trans fats, which have also been shown to significantly reduce inflammation [43]. In this context, several dietary models well recognised for their anti-inflammatory effects stand out. Dietary approaches such as the Mediterranean diet, the DASH diet, the MIND diet, and the Okinawa diet are recognised for their nutrients that have been shown to effectively reduce inflammation.

The Mediterranean diet emphasises the consumption of plant-based foods such as fruits, vegetables, whole grains, legumes, and nuts and seeds. It promotes reducing foods high in saturated fat and including healthy fats, particularly olive oil, which serves as the primary source of fat. Fish and poultry are preferred over red meat, while dairy products such as cheese and yoghurt consumed in moderation. Moreover, moderate wine consumption is permitted, while the intake of sweets and processed foods should be limited [44]. Although the typical Mediterranean diet recommends 1–2 glasses of red wine per day (1 for women and 2 for men) when it’s not contradictory to religious and social forms, an alcohol-free version has come up in recent years [45]. In 2023, the World Health Organization (WHO) declared that no level of alcohol consumption is safe for our health. It can be recommended to consume foods rich in polyphenols (grape, pomegranate bitter etc.) instead of alcohol. Numerous studies have indicated that the Mediterranean diet correlates with reduced expression of proinflammatory pathways and increased expression of neuroprotective pathways [46–48]. Furthermore, extra virgin olive oil, a significant component of the Mediterranean diet, has been shown to decrease astrocyte and microglial cell activation by lowering the production of inflammatory cytokines. Additionally, its extensive antioxidant effects may support the enhancement of brain antioxidant enzymes and diminish the levels of reactive oxygen species (ROS) [49].

The Dietary Approaches to Stop Hypertension (DASH) diet, which highlights high consumption of fruits, vegetables, whole grains, low-fat dairy products, and reduced sodium intake, has also been linked to a slower cognitive decline [50]. Although both (Mediterranean and DASH diets) dietary patterns are beneficial in preventing cognitive decline, neither was originally tailored specifically for brain health. The Mediterranean-DASH Intervention for Neurodegenerative Delay (MIND) diet is a hybrid model that incorporates modifications based on the most compelling evidence in the diet-cognition relationships [50]. It emphasizes limiting foods high in saturated fat while including brain-healthy foods such as green leafy vegetables, berries, extra-virgin olive oil, nuts, whole grains, and low-fat protein sources [51]. The MIND diet rich in neuroprotective nutrients, including, antioxidants, polyphenols, B vitamins, and polyunsaturated fatty acids [52]. The components of the MIND diet comprise 10 brain-healthy food groups (green leafy vegetables, other vegetables, nuts, berries, beans, whole grains, seafood, poultry, olive oil, and wine) and 5 unhealthy food groups (red meat, butter and margarine, cheese, pastries and sweets, and fried/fast food) [53].

The Okinawa diet prioritizes a high intake of vegetables and legumes, especially soy, alongside moderate amounts of fish and alcohol. It features low consumption of meat, dairy, and calories. This diet is abundant in omega-3 fatty acids, maintains a high ratio of monounsaturated to saturated fats, and focuses on carbohydrates with a low glycemic index. It may also be effective in reducing inflammation due to its high phytochemical and antioxidant content [54].

The Healthy Nordic Diet (HND) emphasises the consumption of plenty of fruits, vegetables, whole grains and fish, while limiting the consumption of saturated fat and red and processed meat. While olive oil is the main source of fat in the Mediterranean diet, canola oil is preferred in the HND. In addition, local berries such as blueberries, lingonberries and strawberries play an important role in the HND. The healthy Nordic diet has been shown to have a potential effect on reducing low-grade inflammation [55]. These diets share similar core principles and recommendations. They form part of a holistic lifestyle incorporating regular exercise, social interaction, sufficient sleep, and a diet rich in fresh, seasonal, and locally sourced foods. The key food groups in these diets include abundant fruits and vegetables, with suggestions for at least 5 servings per day. Whole grains and healthy fats, such as olive oil, are highly recommended. Protein primarily comes from legumes and lean sources such as fish and chicken, while red meat is consumed infrequently, about once every 1 to 2 weeks. Importantly, these diets do not significantly trigger inflammatory responses [56].

The putative benefits of Mediterranean, DASH, MIND, Nordic, and Okinawan-style patterns are unlikely to be explained solely by nutrient density. Beyond their overall anti-inflammatory dietary profiles, these patterns are also characterised by a high intake of bioactive food components (e.g., polyphenols and other phytochemicals) that may help support brain health by modulating neuroinflammatory signalling and related oxidative stress pathways [57]. Bioactive food compounds include ingredients with diverse effects in the human body, many of which display antioxidant activity and are linked to reduced inflammation. Accordingly, diets rich in these compounds may help lower oxidative stress and, in turn, attenuate inflammatory responses [57]. Polyphenols, known for their anti-inflammatory and neuroprotective properties, include resveratrol, curcumin, catechins, isoflavones, flavones, flavanones, flavanols, and anthocyanins. These bioactive compounds are associated with numerous health benefits, particularly in protecting neuronal function and reducing oxidative stress [57]. Resveratrol, which is commonly found in grapes, grape juice, red winepeanuts, pistachios, cranberries, berries, and cocoa, has been linked to improved cognitive function and reduced neuroinflammation [58]. Curcumin, a key component of turmeric, exhibits strong antioxidant, anti-inflammatory and neuroprotective effects [59]. Catechins found in green tea, apples, berries, grape seeds, and wine contribute to neuroprotection by modulating oxidative pathways [60]. Isoflavones, abundant in soybeans, chickpeas, lentils, and other legumes, have been shown to exhibit a variety of potential activities, including anti-inflammatory and neuroprotective properties [61]. Flavones found in parsley, thyme, onions, and broccoli, along with flavanones from citrus fruits and tea, play a crucial role in modulating neuroinflammatory pathways. Furthermore, flavanols in cocoa, tea, red wine, grapes, apples, and dark chocolate display potential antioxidant, anti-inflammatory, anti-apoptotic, and immunomodulatory properties [62]. Anthocyanins, abundant in cherries, blackberries, strawberries, and pomegranates, possess strong antioxidative and anti-inflammatory properties, further supporting neuronal integrity. By modulating key signalling pathways, they play a crucial role in preventing neuroinflammation. More importantly, their ability to cross the blood-brain barrier enhances their neuroprotective potential [63]. In light of these findings, incorporating polyphenol-rich foods into the diet could be a promising strategy to mitigate inflammation and support cognitive function [59].

Another bioactive component demonstrated to have neuroprotective effects on brain health is omega-3 fatty acids (e.g., EPA and DHA). These essential fatty acids compose neuronal cell membranes in the brain [64]. Sources of omega-3 include fatty fish, flaxseed (also known as linseed), soybean oil, and canola oil, as well as chia seeds and walnuts. Cold-water fatty fish such as salmon, mackerel, tuna, herring, and sardines are abundant in omega-3 fatty acids. The omega-3 content in fish varies based on their dietary composition. Generally, farmed fish contain higher levels of EPA and DHA compared to wild-caught fish, although this can fluctuate depending on their feed. The National Academy of Medicine (formerly the Institute of Medicine) recommends Adequate Intakes (AIs) for Omega-3: 1.6 g for men and 1.1 g for women [65]. The European Food Safety Authority (EFSA) also advocates for the consumption of 1 to 4 portions of fish per week to protect against the neurodevelopmental toxicity of methylmercury and to ensure the benefits of fish consumption [66]. However, it emphasises that fish and species high in mercury, such as king mackerel, marlin, orange roughy, shark, swordfish, tilefish (Gulf of Mexico), and bigeye tuna should be limited in the diet to meet these targets [65, 66]. Beyond bioactive constituents and overall dietary patterns, certain dietary strategies, such as the ketogenic diet, may influence neurocognitive outcomes by deliberately shifting systemic metabolism towards physiological ketosis, thereby increasing circulating ketone bodies and providing a distinct mechanistic framework [67, 68]. The ketogenic diet operates by substantially decreasing carbohydrate consumption and increasing fat intake, thereby shifting the body’s primary energy source from glucose to ketone bodies synthesised by the liver. When daily carbohydrate intake drops below approximately 20 to 50 g, the consequent reduction in insulin levels induces heightened lipolysis and the liberation of free fatty acids from adipose tissue. These fatty acids are subsequently transported to the liver, where they are converted into ketone bodies, such as β-hydroxybutyrate, acetoacetate, and acetone. In conditions of limited glucose availability, these ketone bodies function as alternative energy sources for the brain, muscles, and other tissues [67, 69]. Ketone bodies have been described as nutritional biomarkers that support antioxidative processes, promote mitochondrial integrity, and support neurosynaptic function [70]. They also function as signalling molecules beyond their role in energy metabolism, influencing gene expression, inflammation, and neurotransmitter balance. For instance, β-hydroxybutyrate (βOHB) inhibits histone deacetylases, thereby engaging epigenetic mechanisms and anti-inflammatory pathways, and has been implicated in initiating anti-inflammatory cascades that counteract the pathology of neurological disease. βOHB accumulation in hippocampal neurons reduces HDAC2/HDAC3 recruitment to the BDNF promoter, thereby increasing BDNF transcription, a key neurotrophic signal that supports learning and memory [71]. In addition to molecular modulation, ketone bodies contribute to the brain’s bioenergy, which could support cognitive abilities. Given that metabolic bioenergy is a primary target in drug development to address cognitive diseases, it is crucial to assess emerging trends in both basic and clinical research on the role of ketone bodies in cognition [70]. The ketogenic diet causes a fundamental shift in metabolism and hormone levels, replacing glucose with ketones as the body’s primary fuel. This change improves energy efficiency, alters cellular signalling, and stabilises neuronal activity mechanisms, supporting its therapeutic benefits in epilepsy, metabolic disease, and emerging investigational uses. The ketogenic diet may influence cognitive function through several pathways, including metabolic switching and fuel substitution, enhanced cellular bioenergetic efficiency, signalling and regulatory effects, and improved neurophysiological stability [67, 71]. These mechanisms are not fully understood but include central nervous system carbohydrate reduction and glycolysis inhibition, changes in neuronal excitability mediated by ATP-sensitive potassium channels through alterations in mitochondrial function, inhibition of the mammalian target of rapamycin (mTOR) pathway, and inhibition of glutamatergic excitatory synaptic transmission [72]. Ketone bodies also provide an efficient alternative fuel for the brain, supporting ATP generation by lowering the NAD⁺/NADH ratio and increasing the coenzyme Q/QH₂ ratio and the ΔG′ of ATP hydrolysis. Beyond serving as substrates, KBs and ketogenic diets (KD) may enhance mitochondrial function by promoting mitochondrial biogenesis via NAD⁺-dependent signalling pathways involving SIRT1/2/3, AMPK, PGC-1α, and FOXO1/3a, and by increasing mitochondrial respiration through PGC-1α–mediated effects on respiratory complexes (e.g., complex I/II). Ketone bodies and ketogenic diets may exert neuroprotective effects by enhancing anti-apoptotic and antioxidant defences (e.g., via inhibition of mPTP opening, mitochondrial ROS production, and caspase activation, and activation of SIRT1/2/3–PGC1α–FOXO1/3a and ERK/CREB/eNOS pathways, with Nrf2 engagement), and by modulating NAD⁺-dependent signalling and epigenetic/post-translational regulation (including HDAC/p300-mediated histone modifications) that can upregulate neurotrophic programs such as BDNF expression [69]. Collectively, these ketone body– and ketogenic diet–driven signalling cascades may strengthen mitochondrial quality control, increase ATP production, enhance anti-apoptotic and antioxidant defences, and thereby contribute to neuroprotection [69]. In neurological conditions, ketogenic interventions have been explored for potential cognitive and neuroprotective effects: a recent systematic review of ten randomised controlled trials reported improvements in cognitive function and brain energy metabolism in Alzheimer’s disease, and another review suggested possible benefits of both ketogenic diets and exogenous ketone supplementation [68, 73]. However, it has also been noted that most existing studies are small, frequently uncontrolled, and primarily evaluate short-term cognitive effects of ketosis; consequently, large, long-term randomised controlled trials examining the impact of ketogenic diets in individuals with cognitive impairment remain lacking and are needed [73]. In epilepsy, ketogenic dietary therapy is an established treatment, particularly for infantile, refractory, and metabolic epilepsies such as glucose transporter type 1 (GLUT1) deficiency and pyruvate dehydrogenase deficiency, where ketosis may reduce seizure frequency and severity, potentially via enhanced mitochondrial efficiency and dampened excitatory synaptic transmission, although mechanisms remain incompletely understood [67, 69, 70, 71]. However, evidence specifically addressing brain fog remains lacking. Notably, “brain fog” has been described as a potential early symptom during the first weeks of a ketogenic diet, according to a study by Skartun et al. (2025) [75]. This initial period of keto-induction is often accompanied by a cluster of transient adverse effects commonly termed the “keto-flu”, including brain fog, headache, lightheadedness, fatigue/lethargy, reduced exercise capacity, mood changes, gastrointestinal symptoms (e.g., constipation or diarrhoea), muscle cramps, and halitosis [74]. In the same study, brain fog and/or reduced cognitive performance were reported by approximately 10% of adults during the ketogenic diet [74]; however, it remains unclear whether these symptoms improve with longer-term adherence, and even one report has described worsening cognitive outcomes [75]. For example, Afzal and Salzman (2024) reported a case of a 48-year-old woman who developed gradually worsening memory loss and brain fog after two years of continuous strict ketogenic dieting; formal cognitive testing showed deficits in attention, anterograde memory, and executive functions, which improved markedly within two months of discontinuing the diet, with repeat testing returning to normal [75]. As a result, mechanistic and molecular studies generally support plausible neuroprotective pathways of the ketogenic diet, yet the available clinical evidence remains limited and heterogeneous. The effect of long-term ketosis/carbohydrate restriction on cognitive function in adults is unclear [67]. Another key limitation concerns compliance and long-term sustainability. Despite potential clinical benefits, the ketogenic diet is difficult to implement in routine practice, and adherence often declines over time because of its restrictive macronutrient targets and substantial planning demands. Sustainability may be further reduced in real-world settings by social occasions, family meals, and cultural eating norms [67]. Notably, the safe and effective implementation of the ketogenic diet typically requires coordinated teamwork among physicians, dietitians, and behavioural/psychological support specialists. Through appropriate patient selection, personalised meal planning with micronutrient adequacy, careful management of the ketosis transition, close monitoring, and ongoing education, such multidisciplinary care may help minimise adverse effects, support adherence, and optimise clinical outcomes [67].

### Gut-brain Axis and Diet

The nervous system connecting the gut and the brain is closely linked. The brain and gut operate within a dynamic communication network that constantly influences one another. The brain regulates gut function through the hypothalamic-pituitary-adrenal (HPA) axis and the autonomic nervous system; neurochemicals like norepinephrine play a critical role in this process. In turn, the gut modulates the central nervous system by producing microbiota-derived metabolites and products, neuroactive substances, peptides, and gut hormones. These signals reach the brain through multiple pathways, such as the enteric nervous system, vagus nerve, circulatory system, or immune responses. These bidirectional interactions converge in a conceptual framework known as the ‘gut-brain axis,’ emphasising the deep interdependence of the two systems [76]. The gastrointestinal tract serves as the body’s largest immune organ, where microbiota and the intestinal mucosal layer collaboratively regulate host immunity [77]. A common symptom of long COVID brain fog is gastrointestinal discomfort, which can present as nausea, abdominal pain, reduced appetite, heartburn, and constipation [78–80]. Alterations in intestinal inflammation, changes in intestinal microbial flora, and cytokine storms may trigger neuroimmune interactions, thereby activating sensory neurons and impacting cognitive function via a neural route [81, 82]. Additionally, changes in the gut microbiome and dysbiosis may contribute to these symptoms [76, 83–85]. Recent research highlights a strong link between gastrointestinal dysfunction and cognitive symptoms, especially brain fog. A clinical study showed that over 50% of patients with common gastrointestinal disorders experienced brain fog, with a higher prevalence noted in individuals diagnosed with gastroparesis and irritable bowel syndrome (IBS) [86, 87]. In the study by Altinsoy and Dikmen, researchers determined that an increase in gastrointestinal symptoms was positively correlated with both brain fog symptoms and the severity of brain fog; they also found that gastrointestinal symptoms significantly predicted brain fog [23]. Brain fog is also commonly reported as a symptom of celiac disease, with a gluten-free diet often recommended as a treatment option [17, 88, 89]. Lichtwark et al. (2014) performed a longitudinal pilot study involving newly diagnosed celiac patients [90]. The study revealed that cognitive impairments, such as diminished attention and motor function, showed significant improvement after 12 months of strict adherence to a gluten-free diet. These improvements correlated with mucosal healing and decreased tissue transglutaminase antibody levels, highlighting the potential reversibility of cognitive deficits in celiac disease with dietary management. Additionally, similar cognitive symptoms have been noted in individuals with non-celiac gluten sensitivity, indicating a wider range of gluten-related neurocognitive effects [88]. These findings suggest that gastrointestinal dysfunction may be a potential trigger rather than a mere concomitant of brain fog symptoms. Therefore, therapeutic interventions targeting the gastrointestinal system may benefit individuals suffering from brain fog symptoms.

**Fig. 1:**
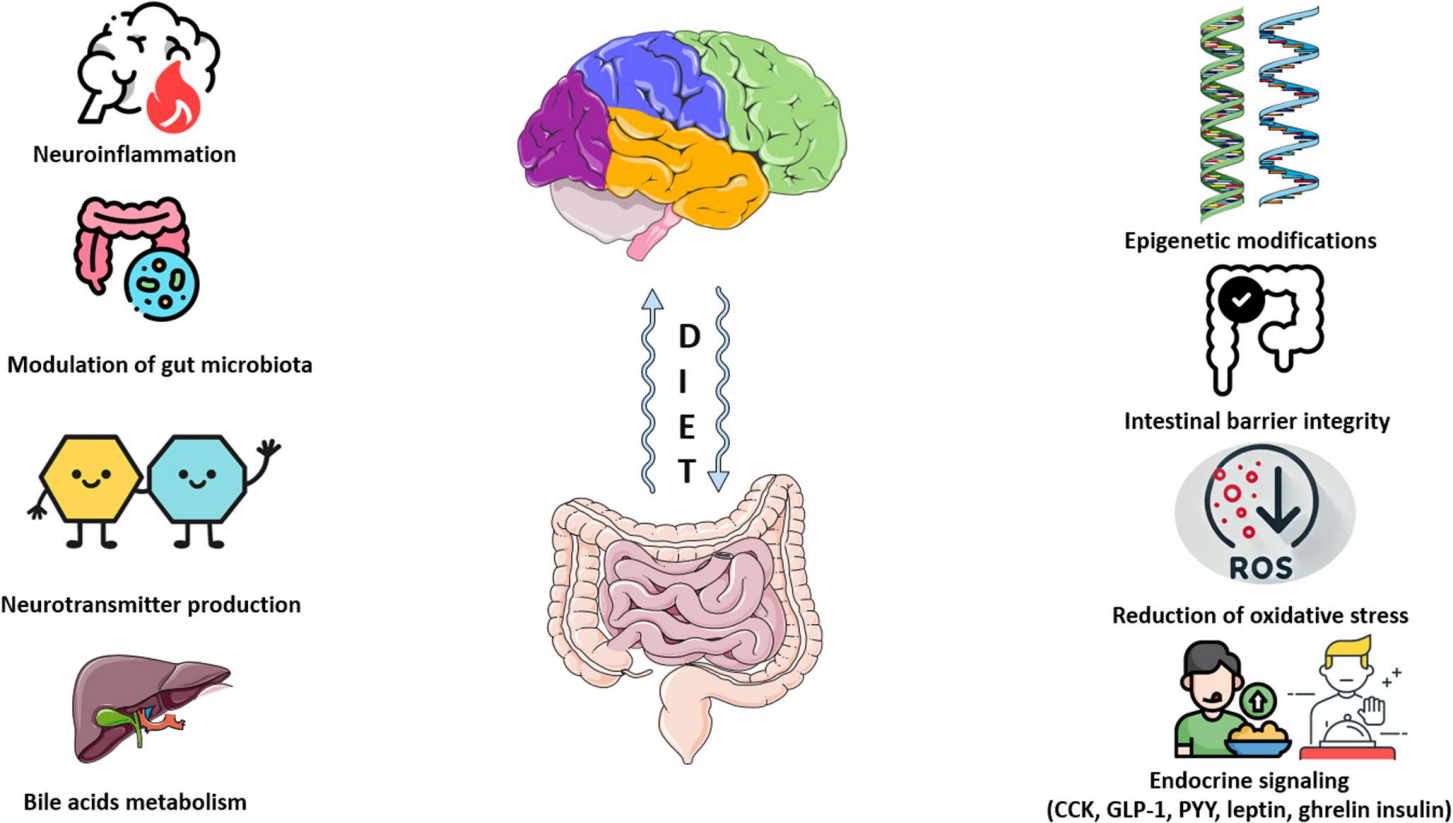
Ways in which diet influences the brain-gut axis. The figure was modified with text, and after adaptation of images from Servier Medical Art by Servier, licensed under a CreativeCommons Attribution 3.0 Unported License (https://smart.servier.com)

Although there is limited data in the literature concerning the role of diet on brain fog and its effects on alleviating symptoms [23], it is understood that diet plays a crucial role in shaping the formation and composition of gut microbiota throughout life. The key mechanisms by which diet modulates the gut–brain axis are summarised in Table 1 and illustrated in Fig. 1.

**Table 1:** Mechanisms of Diet-Induced Gut-Brain Axis Regulation

The mechanisms through which diet influences the gut-brain axis	Description	Dietary components	Physiological effects
**Microbiota Composition and Metabolism**			
Modulation of Microbiota [76] Production of SCFAs [91, 92] Bile Acids (BA) and FXR/TGR5 Receptors[93, 94]	• Diet influences cognitive function by affecting the composition and diversity of the gut microbiota. • Fermentable fibres and resistant starches in the diet are converted to short-chain fatty acids (acetate, propionate, butyrate) by intestinal bacteria. • Diet modulates bile acid–microbiota crosstalk, as dietary components shape gut microbiota composition and function, which in turn determines bile acid metabolism, while bile acids reciprocally influence microbial composition.	• Probiotics • Prebiotics • Synbiotics • Postbiotics • Dietary fiber • Fermented foods • Omega-3 fatty acids • Short chain fatty acids (SCFAs) • Zinc • Resistant starch • Inulin/FOS • Beta-glucans • Pectin • Polyphenols	• Promote the proliferation of beneficial bacteria and improve gut-brain signalling • Enhances gut barrier integrity and regulates permeability. Increases the expression of tight junction proteins (occludin, claudin, zonula occludens). • Strengthens the gut mucus layer via increased Mucin 2 expression. • Modulates oxidative stress by reducing H₂O₂-induced DNA damage and restoring glutathione levels. • Upregulates neurotrophic factors involved in neuronal growth. Induces the expression of tryptophan 5-hydroxylase 1, promoting serotonin biosynthesis. • Diet, probiotics, and prebiotics can regulate bile acid metabolism and the gut microbiome, potentially aiding in developing therapeutic approaches to metabolic and digestive health. • Bile acids function as physiological detergents, essential for the absorption of dietary fat, steroids, and lipid-soluble vitamins, facilitating nutrient uptake. • Beyond their digestive function, bile acids act as signal molecules and endogenous ligands that activate the nuclear farnesoid X receptor (FXR) and membrane Takeda G protein-coupled receptor 5 (TGR5), also known as G protein-coupled bile acid receptor-1. • Integrates nutrient absorption by regulating bile acid metabolism and glucose/lipid metabolism via FXR/TGR5 receptor activation.
**Neural and Hormonal Communication**			
Neurotransmitter Production [95] Vagus Nerve Activation [96, 97] Endocrine Signaling [98 ]	• Gut bacteria produce and/or consume a wide range of neurotransmitters, including dopamine, norepinephrine, serotonin, or gamma-aminobutyric acid (GABA). • The vagus nerve (VN) plays a crucial role in the diet-gut-brain axis by facilitating bidirectional communication between the gut and the brain, sensing dietary components and microbiota metabolites, regulating neurotransmitter release, modulating immune responses, and influencing gut motility, digestion, and metabolic homeostasis. • Diet plays a crucial role in regulating appetite and energy balance through the gut-brain axis, primarily via endocrine signaling.	• Foods rich in tryptophan (dairy products, meat, fish, eggs, bananas, oats, pumpkin and sesame seeds, chocolate, dried dates, soy, tofu, tree nuts, peanuts) • Polyphenols • Choline-rich foods (eggs, red meat, fish) • Fiber-rich foods • Omega-3 fatty acids • Magnesium	• Modulates key neurotransmitter systems (serotonin, GABA, dopamine, norepinephrine) to regulate mood, cognition, reward, and neuronal excitability. • Activates the vagus nerve-mediated cholinergic anti-inflammatory pathway, reducing systemic inflammation and facilitating bidirectional gut-brain communication. • Regulates appetite and energy homeostasis by modulating gut-derived hormones (CCK, GLP-1, PYY), adipokines (leptin), and influencing hormonal and metabolic systems, including ghrelin, the endocannabinoid system, and insulin signaling.
**Barrier Integrity and Systemic Balance**			
Intestinal Barrier Integrity [91] Hypothalamic-pituitary-adrenal (HPA) axis regulation [99]	• Diet can reinforce both the structure and function of the intestinal barrier. • The HPA axis plays a central role in mediating the stress response and regulating the interaction between the gut and the brain. Alterations in the gut microbiota, whether due to diet, antibiotics, or other factors, can impact the stress response, HPA axis activity, and overall cognitive health.	• Prebiotics & probiotics • Omega-3 fatty acids • Polyphenols • Zinc • Glutamine • Vitamin A • Dietary fiber Magnesium • Vitamin C	• SCFAs, produced by the gut microbiota, serve as an energy source for colonocytes and promote immune tolerance through the induction of T regulatory (Treg) cells, thereby contributing to gut barrier integrity and overall gut health. • Diet-derived metabolites activate innate lymphoid cells (ILCs) to produce IL-22, which in turn enhances the intestinal epithelium’s production of mucin and antimicrobial peptides (AMPs), fortifying the gut barrier function. • Cortisol regulates gut function by binding to receptors on epithelial, immune, and enteroendocrine cells and modulates gut microbiota composition by altering gut transit time, intestinal permeability, and nutrient availability. • Cortisol influences brain function by binding to glucocorticoid receptors (GRs) in brain regions such as the hippocampus, amygdala, and prefrontal cortex, affecting cognition, emotion, and stress responses
**Genetic and Cellular Regulation**			
Epigenetic Modifications [100] Oxidative Stress Reduction [101]	• Bioactive nutrients and gut microbiota can alter DNA methylation in the central nervous system (CNS) through the gut–brain axis, playing a crucial role in modulating CNS functions. • Complex gut microbiota microbe-microbe and microbiota-host interactions may also influence the oxidative state of the CNS, directly and indirectly, by interfering both with the level of ROS (endogenous and exogenous) and with the antioxidant system.	• Folate • Vitamin B6 • Vitamin B12 • Vitamin C • Vitamin E • Beta-carotene • Selenium • Phytochemicals & polyphenols • Omega-3 fatty acids • Dietary fiber • Berries • Nuts & seeds • Green vegetables	• Microbial-derived SCFAs act as histone deacetylase (HDAC) inhibitors, potentially influencing gene expression through epigenetic modifications. • By synthesising essential vitamins like B12, B6, and folate, gut microbiota may affect the availability of methyl donors for DNA methylation in the central nervous system (CNS), potentially influencing gene expression related to behavior. • The gut microbiota regulates the oxidative state of the CNS through the production of metabolites, including SCFAs, polyphenols, and vitamins, which act as antioxidants to reduce oxidative stress and support neuronal health. • Microbiota-mediated optimization of dietary energy harvest impacts systemic metabolic health, while its regulation of intestinal and blood-brain barrier permeability limits the CNS’s exposure to inflammatory signals and oxidative stress.

Based on previous studies examining the relationship between the gut-brain axis and cognitive functions [102, 103], unhealthy eating habits and poor diet quality may worsen brain fog symptoms. The Western Diet (WD) is characterised by a high intake of saturated fats (from red meat and high-fat foods), refined sugars (including added sugars and sweetened beverages), refined carbohydrates (such as processed grains), salt, animal products, and industrial oils. It is also marked by the consumption of high-calorie, processed, and packaged foods (such as snacks and fast food) and ultra-processed products (including ready-to-eat meals). In contrast, this diet typically lacks essential nutrients such as fibre, vitamins, minerals, and antioxidants found in fresh fruits and vegetables, whole grains, and legumes [104]. Both human and animal studies have indicated that consuming a Western Diet (WD) is associated with impaired hippocampal-dependent learning and memory function and can lead to cognitive dysfunction [105, 106]. Research shows that the harmful effects of the Western diet on cognitive processes are associated with changes in gut microbiota. Even short-term exposure to this diet reduces the abundance of beneficial bacteria (Bacteroidales, *Akkermansia muciniphila*) while increasing populations of harmful bacteria (*Bilophila* sp.) [106–108]. A Western diet (WD) reduces SCFA levels, potentially impairing neuroprotection, insulin signalling, and intestinal barrier function, and promoting endotoxin-producing bacterial translocation, leading to inflammation and reduced insulin sensitivity [106]. Similarly, a high-fat diet has been shown to induce dysbiosis in gut microbiota, subsequently leading to reduced expression of mucus and tight junction proteins in the colon, triggering local and systemic inflammation [109, 110]. These changes have been reported to contribute to impaired brain function and neurobehavioral alterations, including mood, sociability, learning, and memory [111, 112]. In contrast to the Western diet, the Mediterranean diet has been shown to enhance short- and long-term memory, with certain genus-level bacterial abundances predictive of memory outcomes [113, 114]. The Mediterranean diet (MD) exhibits remarkable diversity, encompassing substantial quantities of fresh vegetables, fruits, legumes, whole grains, nuts, olive oil, and fish, while maintaining a minimal consumption of processed foods and red meat. Notably, this dietary pattern is abundant in dietary fibre, a distinctive feature associated with its health-promoting effects [115]. Recent research underscores the essential role of gut microbiota in mediating these benefits, with particular emphasis on Oscillospira, a genus of gut bacteria, concerning both metabolic and neurological health. The research indicated that the abundance of *Oscillospira* was markedly higher among individuals adhering to a Mediterranean diet than those following a Western diet. Elevated levels of *Oscillospira* were correlated with enhanced insulin sensitivity, reduced plasma concentrations of branched-chain amino acids (BCAAs), and increased ratios of cerebrospinal fluid Aβ42:40, a biomarker indicative of a diminished risk for Alzheimer’s disease. Furthermore, a greater presence of *Oscillospira* was correlated with improved preservation of white matter volume over time, implying a potential neuroprotective benefit of the Mediterranean diet via modulation of gut microbiota [114]. The Mediterranean diet al.so encourages a high intake of fruits and vegetables, which has been demonstrated to enhance microbial diversity, increase potentially beneficial bacteria, and reduce harmful bacteria. Fruits and vegetables are also rich in polyphenols, which have been described to exert a prebiotic-like effect by promoting the growth of symbionts and reducing potential pathogens [116]. These findings suggest that the Mediterranean diet may help preserve cognitive function by modulating the gut microbiota [113, 114].

The ketogenic diet can also cause significant changes in the gut microbiota, which can mediate both beneficial and harmful health effects. The ketogenic diet may influence gut microbiota and, in turn, cognitive processes through several plausible, interrelated mechanisms. These include promoting short-chain fatty acid (SCFA) production, reshaping microbial diversity and community composition, and inducing functional shifts in microbial metabolism, particularly SCFA-related and other energy-regulating pathways. In parallel, ketogenic dietary patterns may modulate immune signalling at the gut interface, supporting intestinal mucosal integrity by enhancing mucosal immune function and attenuating inflammatory infiltration, thereby providing a biologically plausible route through which microbiota-mediated changes could translate into downstream effects on cognitive function [117]. The effect of the ketogenic diet on microbial diversity is complex; while some studies show that it causes a significant decrease in diversity, other studies show that it does not cause any significant change [118–122]. A commonly reported alteration is a decrease in Bifidobacterium, observed in both in vitro and in vivo models and further supported by a systematic review and meta-analysis [119, 120, 123, 124]. *Akkermansia muciniphila* is often reported to increase, and in certain studies this shift has been linked to improved metabolic profiles and enhanced treatment response [118, 119, 123, 125, 126]. Ang et al. demonstrated in their landmark study in this field that metagenomic and metabolomic analyses applied to faecal samples in an eight-week controlled inpatient human study reshaped the microbial community structure and function of the ketogenic diet in a different direction from that of a high-fat diet. Gradient diet experiments in mice confirmed this effect, revealing a reproducible decrease in bifidobacteria under the ketogenic diet; parallel in vitro and in vivo findings supported that ketone bodies selectively suppress bifidobacteria growth. Furthermore, single-species colonisations in germ-free models and human microbiota transplants have demonstrated that the microbial composition accompanying the ketogenic diet reduces proinflammatory Th17 cell levels in the gut, suggesting that ketone-mediated microbiota changes may modulate the mucosal immune response [124]. On the other hand, depletion of beneficial fibre-fermenting taxa under ketogenic diets may be problematic when fibre intake is insufficient over the long term [120, 123]. In parallel, reductions in SCFA-producing bacteria and lower butyrate/acetate concentrations have been reported, although the clinical relevance of these changes is largely inferred from the well-established roles of SCFAs in colonic physiology. Given the limited long-term human evidence, ketogenic diet implementation should be individualised, with adequate fibre provision and overall diet quality tailored to the individual’s clinical context and tolerance [120, 123, 127, 128].

While the diet’s impact on cognitive function through gut microbiota modulation is increasingly evident, it is also important to consider how the broader gut-brain axis can be influenced by other factors, such as probiotic supplementation. Probiotics are widely recognised for their gut health benefits and may offer prophylactic or therapeutic value for cognitive symptoms [83, 85, 129] but recent studies caution against their indiscriminate use, particularly in individuals with preexisting gastrointestinal disorders, which may inadvertently trigger neurological side effects [86, 130, 131]. Emerging research highlights a paradoxical relationship between probiotic use and cognitive symptoms such as brain fog, revealing the gut-brain axis’s complex interplay [86, 130, 131]. A striking example is a 2024 case report involving a 47-year-old commercial airline pilot who developed acute brain fog manifesting as somnolence, impaired concentration, and mental fatigue alongside worsened abdominal distress shortly after initiating an over-the-counter probiotic supplement containing 16 strains. Notably, his symptoms subsided only after discontinuing the supplement and completing antibiotic therapy, suggesting a potential probiotic-induced microbial imbalance [132]. This case underscores the critical need for clinical oversight, especially in high-stakes professions where cognitive performance is nonnegotiable. Collectively, these findings advocate for a personalised, clinically guided approach to probiotic supplementation. Healthcare provider consultation is essential to mitigate the risk of exacerbating symptoms, particularly in vulnerable populations [133]. Future research should aim to elucidate the underlying mechanisms of probiotic-induced brain fog and establish evidence-based guidelines for safe and effective supplementation.

### Sleep and Diet

Sleep disturbances are prevalent among individuals experiencing brain fog, characterised by cognitive impairments such as memory loss, reduced concentration, and mental fatigue [134]. Sleep deprivation and poor sleep quality are among the most significant triggers frequently associated with brain fog [18, 135–137]. Krishnan (2022) suggests providing appropriate treatment options for insomnia or poor sleep hygiene in the management of brain fog [8]. A recent study also found a statistically significant negative correlation between brain fog symptoms and sleep quality scores, suggesting that poorer sleep quality may exacerbate brain fog symptoms [23]. This section of the paper will discuss potential dietary approaches that may be beneficial for improving sleep in individuals suffering from brain fog. To better contextualise studies exploring the relationship between sleep and nutrition, it is important first to define key terms essential for understanding sleep health. Sleep duration refers to the total amount of time an individual spends asleep within 24 h. It is a key component of sleep health and varies significantly by age, lifestyle, and individual needs. The National Sleep Foundation considered 7 to 9 h appropriate for young adults and adults and 7 to 8 h for older adults [138]. Sleep quality refers to how restorative, uninterrupted, and refreshing your sleep is, regardless of duration. It focuses on the effectiveness of sleep rather than just the amount of time spent asleep. Even if you meet recommended sleep durations, poor sleep quality can leave you tired, irritable, or unwell, and poor sleep quality contributes to disease and poor health outcomes [138–139]. The objective evaluation of an individual’s sleep quality has been examined in the literature through polysomnography and actigraphy. Polysomnography is the gold standard for evaluating sleep by using physiologic data. Actigraphy is a noninvasive and inexpensive method of monitoring an individual’s periods of rest and activity [140]. By using objective measures of sleep, sleep quality can be characterised by the amount of slow-wave sleep (SWS) and rapid eye movement (REM) sleep a person obtains during the night. Mammalian sleep comprises two basic stages: cyclically alternating SWS and REM sleep. In human nocturnal sleep, SWS predominates initially and decreases in intensity and duration throughout the course of sleep. In contrast, REM sleep becomes more intense and extensive towards the end of the sleep period. SWS is considered deep sleep and serves a restorative function, while both REM and SWS are involved in memory consolidation [141]. Sleep quality is also assessed by the Pittsburgh Sleep Quality Index (PSQI) questionnaire, which is widely used as a subjective assessment tool [142]. Sleep quality encompasses sleep latency (falling asleep within 15–30 min), sleep continuity (minimal awakenings, ideally ≤ 1–2 times per night), sleep architecture (balanced progression through sleep stages), and morning refreshment (waking up feeling restored and energised) [139]. Sleep-related impairment (SRI) refers to cognitive, emotional, and functional disturbances caused by insufficient or low-quality sleep, including daytime fatigue, decreased concentration, and diminished work performance [143].

Emerging evidence highlights a robust association between dietary patterns and sleep health. Systematic reviews and observational studies consistently demonstrate that healthier diets, such as the Mediterranean diet (MD), plant-based regimens, and high-quality dietary patterns, are positively linked to improved sleep outcomes. Alibabaei et al. (2021) conducted a systematic review of 14 studies, encompassing cross-sectional, cohort, and clinical trial designs, to investigate the relationship between dietary patterns and sleep [144]. The findings indicated that longer sleep duration was consistently associated with healthier dietary patterns, such as those rich in vegetables and healthy proteins. Similarly, a systematic review by Arab (2024) synthesised findings from 37 observational studies, encompassing a total sample of 591,223 individuals, and reported that adherence to healthy dietary patterns—such as the Mediterranean diet and high-quality diets—was associated with a lower prevalence of insomnia symptoms [145]. These findings are further supported by Godos et al. (2021), who emphasised that diets rich in plant-based foods, seafood, and low in processed foods and sugar are linked to better sleep quality [146]. In a longitudinal analysis of young adults, increases in fruit and vegetable (FV) consumption were associated with improvements in insomnia symptoms, sleep quality, and time to fall asleep, particularly in women. After 3 months, women who increased FV intake by 3 or more servings experienced significant improvements in insomnia-related sleep difficulties compared to those with no change or a decrease in FV intake [147]. A cohort study by Jansen et al. (2020) examined the relationship between dietary patterns and sleep timing and duration over a two-year follow-up period [148]. The findings revealed that following a plant-based and lean protein diet was associated with earlier sleep timing and a decreased phase delay in sleep timing. Among the dietary patterns recognised as beneficial for sleep health, the Mediterranean diet stands out as a significant example. Several studies have highlighted the positive relationship between adherence to the Mediterranean diet (MD) and sleep quality [149–151]. Campanini et al. (2017) reported that following an MD pattern was associated with a lower risk of changes in sleep duration and better sleep quality [149]. Similarly, a systematic review by Godos et al. (2024) found a significant association between higher adherence to the MD and a reduced likelihood of poor sleep quality, inadequate sleep duration, excessive daytime sleepiness, and insomnia symptoms [150]. Moreover, Scoditti et al. (2022) highlighted that the positive effects of the MD on sleep are likely a result of the synergistic interactions among its various nutrients, rather than the effects of individual elements [151]. A cross-sectional study by Huang et al. (2024) involving 7,987 adults from suburban Shanghai discovered that following healthier dietary patterns, such as the DASH diet and MD, was linked to a lower incidence of poor sleep. Conversely, higher intake of sugar-sweetened beverages and juice was associated with poorer sleep quality [152]. Similarly, unhealthy dietary patterns are strongly associated with sleep disturbances [146, 153–158]. Diets high in ultra-processed foods, refined carbohydrates, and added sugars are linked to poor sleep quality and/or short sleep duration [146, 153]. Makowski (2021) also noted that diets high in saturated fats and sugars correlate with elevated sleep-related impairment scores [139]. Godos et al. (2021) and Delpino et al. (2023) identified processed foods and sugary beverages as key disruptors of sleep architecture, likely due to their pro-inflammatory properties and effects on blood glucose fluctuations [150, 153]. For example, Khorasaniha et al. (2022) found that low fruit/vegetable, high-protein diets increase sleep disorder risks [154], while Gangwisch (2020) highlighted the role of high glycemic index diets in insomnia [159]. In line with these findings, in the prospective Seniors-ENRICA cohort (*n* = 1,341 adults aged ≥ 60 years; 2012–2015), higher habitual meat intake was associated with poorer sleep outcomes; each 100 g/day increment was linked to higher odds of the combined endpoint (≥ 2 h change in sleep duration plus poor sleep quality; OR 1.60, 95% CI 1.07–2.40) in models adjusted for sociodemographic factors, lifestyle, morbidity, and baseline sleep characteristics, with directionally similar findings for red/processed and white meat and stronger associations among those with physical impairment [155]. Because higher meat intake may co-occur with other sleep-relevant exposures (e.g., alcohol intake and caffeinated sugar-sweetened beverages [SSBs], such as cola) and with more processed/fast-food dietary patterns, residual confounding and dietary co-patterning cannot be fully excluded [155].

Evidence linking macronutrient distribution to sleep remains mixed, with effects varying by population and by the specific sleep domain assessed. Tanaka et al., in 4,835 Japanese civil servants, reported domain-specific associations between macronutrient energy percentages and insomnia symptoms assessed by three brief items: difficulty initiating sleep (trouble falling asleep), difficulty maintaining sleep (nighttime awakenings), and poor sleep quality. Low protein intake (< 16% energy) was linked to poorer sleep quality (and borderline more trouble falling asleep), whereas higher protein (≥ 19% energy) and low carbohydrate intake (< 50% energy) were each borderline associated with more nighttime awakenings; fat intake showed no clear association after adjustment. They suggested tryptophan-related pathways (competition with other large neutral amino acids and insulin-mediated shifts after carbohydrate intake) as possible mechanisms, but the cross-sectional design and brief symptom items preclude causal inference or conclusions about clinical insomnia [156]. At the extreme low-carbohydrate end, evidence from ketogenic dietary therapies (KDTs) remains variable and is often derived from heterogeneous designs in which sleep is not the primary endpoint. In their scoping review, Pasca et al. conducted a web-based search of PubMed and Scopus up to June 2023 (277 records screened) and included 20 studies, highlighting substantial variability in study designs and sleep outcome assessments. Overall, KDTs were associated with mixed but sometimes favourable changes in subjective sleep domains (e.g., overall sleep quality, sleep initiation, nighttime awakenings, and daytime sleepiness), with some reports also noting alterations in sleep structure such as increased REM sleep; however, the heterogeneity and non-primary sleep assessments limit firm conclusions [160]. Providing objective, experimental insight, Afaghi et al. used polysomnography to assess the acute effects of an Atkins-like very-low-carbohydrate (VLC; <1% energy from carbohydrates, 61% fat, 38% protein) diet in 14 healthy, non-obese young men, both after a single VLC evening meal and after 48 h of VLC dieting (ketosis phase). Compared with the control night, VLC conditions increased slow-wave sleep (SWS; stages 3–4), decreased REM sleep, and increased the arousal index during light NREM sleep (stages 1–2); notably, these changes also occurred before ketosis, suggesting effects driven by macronutrient-composition–related physiological signals rather than ketosis per se [161]. In a systematic review and meta-regression, Sutanto et al. (2020) concluded that, in healthy adults, the overall evidence tends to favor a higher proportion of energy from protein in relation to better sleep quality. Across included studies, “good sleepers” (typically defined by ≥ 7 h sleep, PSQI ≤ 5, sleep latency ≤ 30 min, and sleep efficiency ≥ 85%) generally consumed diets with relatively higher protein and lower carbohydrate and fat contributions, a pattern the authors noted may be more pronounced in individuals with obesity. However, meta-regression based on a limited number of eligible studies did not show a clear dose–response between macronutrient percentages and sleep duration, and the authors emphasized limitations including reverse causality (predominantly cross-sectional data) and possible effect modification by macronutrient source/type (e.g., fatty acid profile, carbohydrate glycaemic characteristics, protein source) [162]. Focusing on carbohydrates, Vlahoyiannis et al. conducted a systematic review with meta-analysis and meta-regression of clinical trials assessing carbohydrate quantity, quality (GI/GL), and timing in relation to objectively measured sleep outcomes (polysomnography/EEG and, in some studies, actigraphy). Across 11 studies (27 trials; 16 comparison datasets), lower versus higher carbohydrate intake was associated with a moderate increase in N3 (slow-wave) sleep, whereas higher carbohydrate intake was linked to a moderate increase in REM sleep—suggesting carbohydrate quantity may shift sleep architecture even when global sleep indices change little. Carbohydrate quality showed no clear effect on sleep-stage distribution, although meta-regression suggested that the magnitude of carbohydrate manipulation and changes in glycaemic load partly explained heterogeneity in sleep initiation and maintenance metrics (e.g., sleep onset latency, sleep efficiency, wake after sleep onset) [163]. Taken in a wider dietary context, Wilson and colleagues synthesised 20 human studies (6 observational, 14 interventional; 1975–March 2021) examining diet composition and objectively assessed sleep (polysomnography/EEG or actigraphy). Associations for carbohydrate and fat quantity were inconsistent, whereas macronutrient quality emerged as a key modifier: higher fiber/complex carbohydrates and healthier (unsaturated) fats were more consistently linked to better objective sleep profiles, while higher saturated fat and lower fiber patterns were associated with poorer indices in several interventions. Higher protein intake was generally associated with more favourable sleep outcomes, although effects may vary by context and baseline sleep status [164]. Collectively, these findings suggest that macronutrient balance may influence sleep architecture; however, actionable implications likely lie less in macronutrient percentages per se and more in overall diet quality and whole-diet patterns (e.g., Mediterranean-style, fiber- and plant-rich, lower in saturated fat and added sugars) [157]. Longer-duration trials in free-living settings using whole foods, objective sleep endpoints, and mechanistic measures are needed to clarify causality and clinical relevance.

**Fig. 2:**
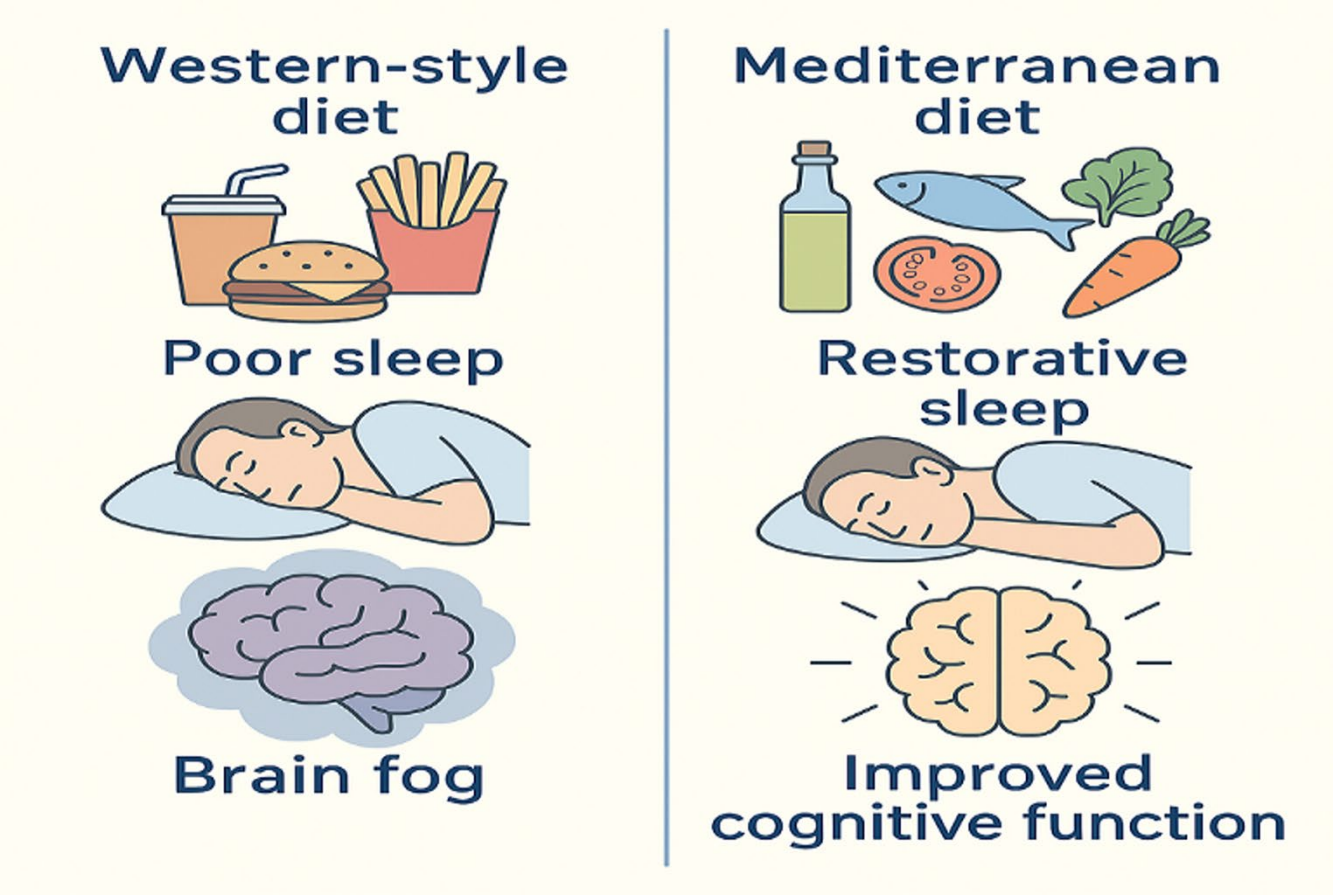
Differential Effects of Diet and Sleep on Brain Fog: Mediterranean Patterns and Restorative Sleep May Protect, While Western Diets and Poor Sleep May Worsen Symptoms

Diet quality and composition are important; however, the timing of dietary intake may be equally critical. Despite this, its impact on sleep remains largely unexplored. Emerging evidence suggests that chrononutrition, which considers not only what is eaten but also when and how often food is consumed, can influence metabolic processes and overall health by entraining the body’s circadian clock. Accordingly, meal timing and eating patterns may play a significant role in sleep health [165–167]. The study conducted by Kim et al. using NHANES 2017–2020 data aimed to identify “chrononutrition profiles” based on meal timing and patterns among US adults (n = 5228) and to test their association with different dimensions of sleep health (particularly sleep timing, duration, regularity, and total “sleep health score”). Latent profile analysis using chrononutrition indicators identified five distinct eating timing patterns: Typical Eating (reference/ “average” pattern), Early Finished Eating (ending the day earlier), Later Heavy Eating (shifting the eating load to later hours/heavier in the evening), Extended Window Eating (wide eating window), Restricted Window Eating (narrow eating window) were defined, and their relationships with dimensions such as sleep timing, duration, and regularity were tested. In the adjusted models, the lengthening of the wake-to-first-meal interval was consistently associated with more unfavourable sleep timing and the likelihood of inadequate sleep duration; the “later heavy eating” profile was associated with poorer sleep timing, while the “restricted window eating” profile was associated with poorer sleep duration [168]. Chung et al., in their analysis based on online survey data from 793 young adults, reported that eating behaviour within 3 hours before bedtime was associated with nighttime awakenings (adjusted OR ≈ 1.4); however, they did not find a significant association with sleep onset latency > 30 min or short sleep duration (≤ 6 h) [169]. Bedtime eating behavior is associated with sleep outcomes in different populations. In the Hong Kong sample (n = 215), nighttime eating tendency was positively associated with poor sleep quality and higher dream intensity; although this relationship was moderated by sleep quality and related beliefs, it was not fully explained by them [170]. In the American Time Use Survey data (2003–2018) representing the United States, eating/drinking less than 1 hour before bedtime appears to be associated with longer sleep duration while also increasing the likelihood of wake after sleep onset (WASO); as the time of eating/drinking moves further away from bedtime, the likelihood of WASO and extreme sleep durations decreases [171]. In a prospective female cohort (n ≈ 28,000), the tendency to eat at conventional times decreased with shorter sleep, while snacking increased with shorter sleep and was accompanied by more unfavourable dietary patterns. Together, these findings suggest that late-night eating may particularly disrupt sleep continuity, while extended sleep duration may be a compensatory response to impaired sleep efficiency in some individuals; however, stronger designs are needed to establish causality [172]. Because late-night eating often co-occurs with irregular eating patterns throughout the day, it is also important to consider meal skipping as another potentially modifiable correlate of sleep. Some studies have shown that skipping breakfast (and in some studies, lunch) may be associated with poorer sleep quality or shorter sleep duration [158, 173, 174]. Collectively, the evidence suggests that optimising meal timing (avoiding intake close to bedtime) alongside maintaining regular daytime meals may represent pragmatic, modifiable targets to support sleep health, although causal relationships remain to be confirmed. More broadly, the diet–sleep relationship appears complex and likely bidirectional. While healthier dietary patterns and eating habits may support sleep, diets characterised by higher intakes of ultra-processed foods and overall poorer diet quality have been associated with less favourable sleep outcomes (Fig. 2).

When discussing the relationship between nutrition and sleep, certain sleep-promoting foods stand out due to their potential benefits. Among these are foods rich in tryptophan or melatonin, which are particularly noteworthy, as experimental data suggest that their consumption can enhance sleep quality [175]. Tryptophan is an essential amino acid found primarily in animal products, including beef, lamb, chicken, and dairy, as well as nuts, seeds, whole grains, and legumes. Tryptophan, mainly transported in circulation bound to albumin, plays a crucial role in the production of serotonin and the biosynthesis of melatonin. The transport across the blood–brain barrier is regulated by the ratio of free to bound tryptophan, and is affected by competition with other large neutral amino acids (LNAAs) that share the same transport system. As LNAAs become more prevalent in the diet, the availability of tryptophan for cerebral uptake is limited. However, insulin release promotes peripheral protein synthesis, which reduces LNAA competition and facilitates the transport of tryptophan into the brain. Consequently, co-consuming carbohydrates with tryptophan-rich foods may enhance tryptophan levels. In humans, melatonin is synthesised exclusively from dietary tryptophan, although only a small fraction (1–2%) is converted via the serotonin pathway. Within the brain, tryptophan is initially transformed into serotonin, which is then converted into melatonin through enzymatic reactions requiring B vitamins and magnesium as cofactors. Once synthesised in the pineal gland, melatonin is released into circulation. Both endogenously produced and dietary melatonin are rapidly metabolised, primarily in the liver, and are excreted in urine as 6-sulfatoxymelatonin. Given the processes involved in the biosynthesis of serotonin and melatonin in the body, it is biologically plausible that diet can influence sleep quality [176, 177].

Tart cherries and kiwifruits have been extensively studied for their role in sleep regulation, primarily due to their content of melatonin, serotonin, and antioxidants [178–180]. Research has indicated that tart cherries and kiwifruits may enhance sleep quality and duration [178–180]. Tart cherries, particularly in the form of juice, have been investigated for their potential to alleviate sleep disturbances. A randomised, double-blind, crossover study by Pigeon et al. (2010) investigated the effects of a proprietary blend of tart cherry juice on sleep continuity in older adults who have chronic insomnia. They observed significant reductions in insomnia severity and wake time after sleep onset compared to placebo, although no substantial improvements were noted in sleep latency, total sleep time, or sleep efficiency [178]. Likewise, Garrido et al. (2010) evaluated the effects of Jerte Valley cherry cultivars on sleep-wake patterns and markers of oxidative stress. Their results showed increased melatonin levels, enhanced total antioxidant capacity, and improved sleep parameters, indicating that cherries could act as a potential nutraceutical intervention for sleep regulation [179]. Kiwifruit has also been linked to improved sleep outcomes. Lin et al. (2011) conducted a study in which participants consumed two kiwifruits one hour before bedtime for four weeks. The results indicated significant improvements in subjective and objective sleep measures, including reduced sleep onset latency and wake time after sleep onset, alongside increased total sleep time and sleep efficiency [180]. These effects were attributed to the fruit’s high antioxidant and serotonin content, which may influence sleep regulation. These studies suggest that natural dietary sources, such as tart cherries and kiwifruits, may offer a promising, non-pharmacological approach to improving sleep quality. However, further large-scale, controlled trials are needed to confirm their efficacy and elucidate the underlying mechanisms of action.

Recent scientific studies highlight the potential of nuts as natural interventions to improve sleep quality [181, 182]. A study conducted among medical students at Tehran University of Medical Sciences found that consuming sweet al.monds for two weeks significantly reduced the prevalence of insomnia. Before the intervention, 77.6% of participants had insomnia, while 22.4% had normal sleep. After the intervention, 69.2% of participants had insomnia, and 30.8% had normal sleep. The results suggest that sweet al.monds may be beneficial in improving sleep quality [181]. A randomised cross-over trial involving 80 young adults (85.5% women) investigated the impact of daily walnut consumption on sleep quality. Participants either consumed 40 g of walnuts daily or refrained from eating walnuts or other nuts for 8 weeks, with a washout period of 2 weeks. The study found significant improvements in sleep quality, including reduced sleep latency, higher sleep efficiency, and less daytime sleepiness. Additionally, urine samples collected from 20:00 to 23:00 showed a significant increase in the melatonin metabolite 6-sulfatoxymelatonin following walnut consumption. These findings suggest that walnuts, through their melatonin content [183], can improve sleep quality and reduce daytime sleepiness in healthy young adults [182].

Dairy consumption may enhance sleep quality by increasing endogenous melatonin production, largely due to the high tryptophan content. Besides these direct effects, fermented dairy products could further promote better sleep by positively affecting gut microbial composition, thereby fostering serotonin production and, ultimately, melatonin synthesis [184]. Supporting this, both randomised controlled trials (RCTs) and non-RCTs have shown that fermented milk can improve sleep efficacy and reduce wake episodes [185, 186]. Beyond the type of dairy product consumed, the quantity of milk intake has also been linked to sleep outcomes. For example, studies have identified that higher milk consumption is positively associated with longer sleep duration in girls, whereas low milk intake has been correlated with delayed sleep onset in university students [187–190]. Moreover, insufficient calcium intake has been associated with difficulties in falling asleep and experiencing non-restorative sleep in adults. Finally, among older adults, dairy consumption combined with physical activity appears to lead to better sleep outcomes, particularly by reducing subjective sleep latency [190].

Studies on sleep and nutrition have generated considerable interest in how fish consumption affects sleep quality. Emerging evidence suggests that fish, particularly oily varieties rich in omega-3 fatty acids and vitamin D, may improve sleep duration and quality across diverse populations. A longitudinal study by Tani et al. (2022) established a robust association between the consumption of oily fish and improved sleep quality in a healthy Japanese cohort [191]. Participants with a higher intake reported longer sleep durations and more restorative sleep, outcomes the authors attribute to the anti-inflammatory properties of omega-3 fatty acids. These findings support broader hypotheses connecting omega-3s to the neural regulation of sleep-wake cycles. Liu et al. (2017) further emphasise the mediating role of sleep in cognitive outcomes [192]. Their research revealed that regular fish consumption was associated with improved sleep efficiency, enhancing cognitive performance. This suggests that dietary interventions to improve sleep could yield additional benefits for cognitive health. Importantly, interventional studies within clinical populations support these findings. A randomised controlled trial conducted by Hansen et al. (2014) demonstrated that male forensic patients who consumed Atlantic salmon thrice weekly during the winter months exhibited significantly improved sleep latency, efficiency, and daytime functioning compared to a control group [193]. At the population level, insufficient fish intake may indicate broader dietary deficiencies that adversely affect sleep. Hemiö et al. (2020) identified low fish consumption as a component of unhealthy dietary patterns associated with poor sleep quality [194].

### Limitations

This review is limited by the scarcity of studies that specifically examine nutritional exposures or interventions in individuals reporting brain fog as a primary outcome. Therefore, we draw largely on adjacent literatures that support plausible mechanistic links, namely, neuroinflammation, gut–brain axis dysregulation, and sleep disruption, while recognising that these fields often lack consistent definitions and measures of “brain fog”. Overall, this review should be viewed as hypothesis-generating, mapping candidate pathways and highlighting priorities for well-designed intervention studies to test these mechanisms and strengthen causal inference. Future research should prioritise (i) consensus definitions and standardised measurement of brain fog (including both patient-reported outcomes and objective cognitive testing), (ii) adequately powered randomised trials evaluating whole-diet patterns and targeted components, and (iii) mechanistic endpoints linking diet to symptoms through inflammatory markers, barrier integrity, sleep physiology, and microbiome function (metabolomics/SCFAs), ideally within longitudinal designs. Until such data are available, dietary guidance should emphasise nutritionally adequate, anti-inflammatory whole-diet patterns and individualised dietetic support, while avoiding overinterpretation of preliminary evidence on supplements.

## Conclusion and Future Perspectives

Brain fog has emerged as a major cognitive concern in recent years and is reported by patients in the clinic quite frequently, leading to a growing body of research. The underlying mechanisms are often attributed to neuroinflammation, disruption of the gut-brain axis, and reduced sleep quality and duration, which are frequently cited as triggers of brain fog. Although the literature specifically addressing diet and brain fog symptoms is quite limited, dietary interventions are well established in their ability to modulate these fundamental pathways. The Mediterranean diet, in particular, stands out for its robust evidence base supporting its role in reducing neuroinflammation, supporting gut–brain communication, and improving sleep quality. Referring individuals suffering from brain fog to a dietitian during this process is very important in terms of individual approach in nutrition management. A nutritionally balanced diet, adequate in energy and micronutrients and rich in dietary fibre, antioxidants and omega-3 fatty acids, may benefit the management of brain fog. Importantly, dietary strategies represent a non-pharmacological, low-risk approach that may complement existing therapeutic approaches, underscoring the value of interdisciplinary collaboration among nutrition, neurology, gastroenterology, and psychology.

At the population level, addressing brain fog through nutrition and lifestyle modification may have broader implications for mental health, work productivity, and overall quality of life. However, future research must overcome significant methodological challenges, including the lack of standardised diagnostic criteria for brain fog and the need for rigorous, large-scale randomised controlled trials. Longitudinal studies incorporating objective biomarkers (e.g., inflammatory cytokines, gut microbiota profiles), standardised cognitive assessments, and robust measures of nutritional status are particularly needed. In conclusion, while the field remains in its early stages, dietary strategies targeting neuroinflammation, the gut–brain axis, and sleep quality represent a promising frontier in the management of brain fog. Further research in this area could improve clinical care by informing evidence-based dietary interventions and could also support public health policies aimed at strengthening cognitive resilience in today’s society.

## Key References

- McWhirter L, Smyth H, Hoeritzauer I, Couturier A, Stone J, Carson AJ. What is brain fog? J Neurol Neurosurg Psychiatry. 2023;94:321–5. doi:10.1136/jnnp-2022-329683. [of importance]
  - o This study is of outstanding importance as it provides one of the first phenomenological analyses of “brain fog” through first-person social media narratives, highlighting its multidimensional cognitive and emotional components and offering insight into underlying pathophysiological mechanisms.
- Denno P, Zhao S, Husain M, Hampshire A. Defining brain fog across medical conditions. Trends Neurosci. 2025;48:330–48. doi:10.1016/j.tins.2025.01.003. [of outstanding importance]
  - o This review provides a comprehensive roadmap for future research on brain fog, emphasizing the development of harmonised cognitive protocols, validated measurement tools, and biomarker-based stratification.
- Krishnan K, Lin Y, Prewitt K-RM, Potter DA. Multidisciplinary Approach to Brain Fog and Related Persisting Symptoms Post COVID-19. J Health Serv Psychol. 2022;48:31–8. doi:10.1007/s42843-022-00056-7. [of outstanding importance]
  - o This study provide one of the first practical frameworks emphasizing a multidisciplinary and lifestyle-oriented approach to the assessment and management of brain fog. Their model integrates cognitive, affective, sleep, and behavioral factors and highlights the importance of coordinated referrals and individualized care strategies.
- Minoretti P. Clear Skies, Cloudy Mind: Probiotic-Related Brain Fogginess in a Commercial Airline Pilot. Cureus. 2024;16:e66426. doi:10.7759/cureus.66426. [of importance]
  - o This case report is among the first to document probiotic-associated brain fog, suggesting a gut–brain axis mechanism linking gastrointestinal dysbiosis to cognitive symptoms. The clear temporal link between probiotic use and symptom onset, with rapid recovery after antibiotic therapy, highlights the need for cautious probiotic use, particularly in safety-sensitive professions.

## Data Availability

No datasets were generated or analysed during the current study.
